# Implication of xanthine oxidoreductase in oxidative stress-related chronic diseases

**DOI:** 10.3389/fendo.2025.1662037

**Published:** 2026-02-11

**Authors:** Kendra L. Nelson, Venkata Saroja Voruganti

**Affiliations:** 1 Department of Nutrition, University of North Carolina at Chapel Hill, Chapel Hill, NC, United States; 2 Nutrition Research Institute, University of North Carolina at Chapel Hill, Kannapolis, NC, United States

**Keywords:** purine metabolism, uric acid, xanthine, adenosine, oxidative stress

## Abstract

Xanthine oxidoreductase (XOR) catalyzes the final steps of purine catabolism: the oxidation of hypoxanthine to xanthine, and xanthine to UA. XOR exists as interconvertible proteoforms, xanthine dehydrogenase (XDH) and xanthine oxidase (XO). The balance between XDH and XO determines whether purine degradation is redox-neutral or strongly pro-oxidant. Evidence across cardiovascular, renal, oncological and neurological disorders shows that excess XO-derived ROS, rather than UA itself, is a likely mediator of tissue injury and clinical progression, with several lines of research linking them to the interplay between XOR and purinergic signaling. Pharmacological inhibition or down-regulation of XO ameliorates pathology even when UA concentrations remain unchanged, underscoring the therapeutic relevance of the proteoform-specific mechanism. This mini-review focuses on the structure, regulation, and pathological roles of XOR, with emphasis on its implications in oxidative stress-related diseases.

## Introduction

**Figure 1:**
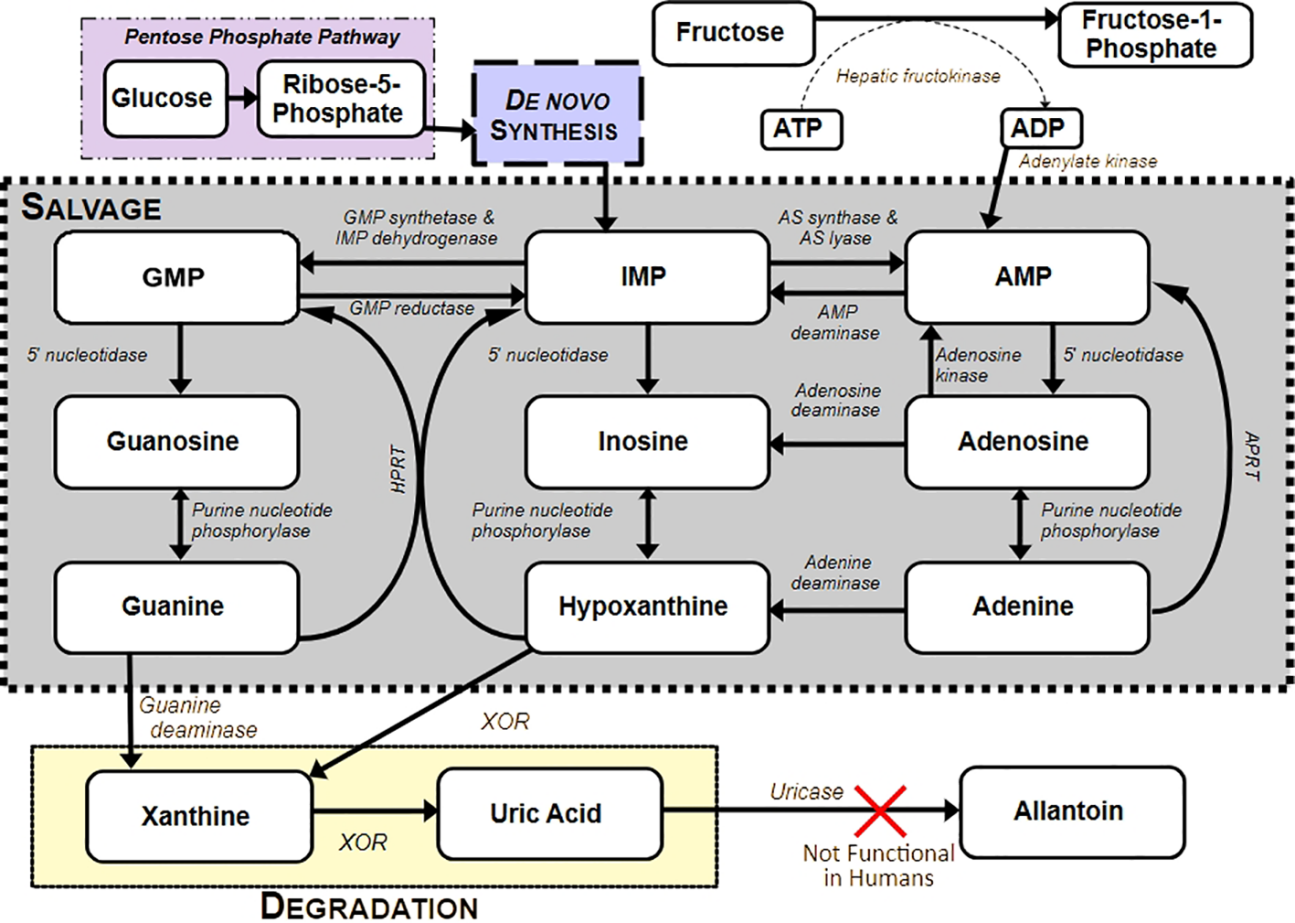
Overview of purine metabolism. An overview of the purine metabolism pathway in humans, showing metabolites and enzymes involved in *de novo* synthesis, salvage, and degradation. ATP, ADP, AMP, Adenosine tri-, di-, mono phosphate; IMP, Inosine monophosphate; GMP, Guanosine monophosphate; XOR, Xanthine oxidoreductase; AS – HPRT, Hypoxanthine-guanine phosphoribosyltransferase; APRT, Adenine phosphoribosyltransferase.

Purine metabolism is essential for cellular energy balance, nucleic acid turnover, and redox regulation (1, 2). In humans, this pathway encompasses *de novo* synthesis, salvage, and degradation, culminating in the sequential oxidation of hypoxanthine to xanthine and then to uric acid (UA) (**Figure 1**) (3). In most other mammals, the enzyme uricase further oxidizes UA into allantoin, though humans lost functional uricase in a series of mutations which occurred between 13–24 million years ago (4–6). This has resulted in humans having a serum urate (soluble ion of UA) concentration approximately five times greater than observed in most other animals (6). This elevated UA concentration may be of great evolutionary importance, as UA primarily functions as an antioxidant when in circulation (7), and it has been hypothesized to be a driver of the large brains and extended life spans of *Homo sapiens* compared to other mammals which maintain functional uricase (8). UA accounts for about 60% of human body’s antioxidant activity, and neutralizes reactive oxygen and nitrogen species and thus, can confer anti-inflammatory and neuroprotective effects (9).

However, research has shown that elevated UA does not always confer benefits. Gout (10, 11), hypertension and cardiovascular disease (CVD) (12, 13), and renal disease (12) are all associated with elevated UA. In pathologies for which UA has been shown to be protective, such as Parkinson’s disease (12, 14, 15) and Multiple Sclerosis (16), associations are not always consistent—evidence exists for both positive and negative associations between UA and these pathologies and others such as cognitive decline (17–19). To explain the lack of consistency, some have focused on the dual role of UA. While primarily considered an antioxidant, UA may serve as a pro-oxidant in some physiological contexts (7, 20). Importantly, the oxidative potential of UA production depends not only on UA itself but on the enzyme responsible for its formation: xanthine oxidoreductase (XOR) (3).

**Figure 2:**
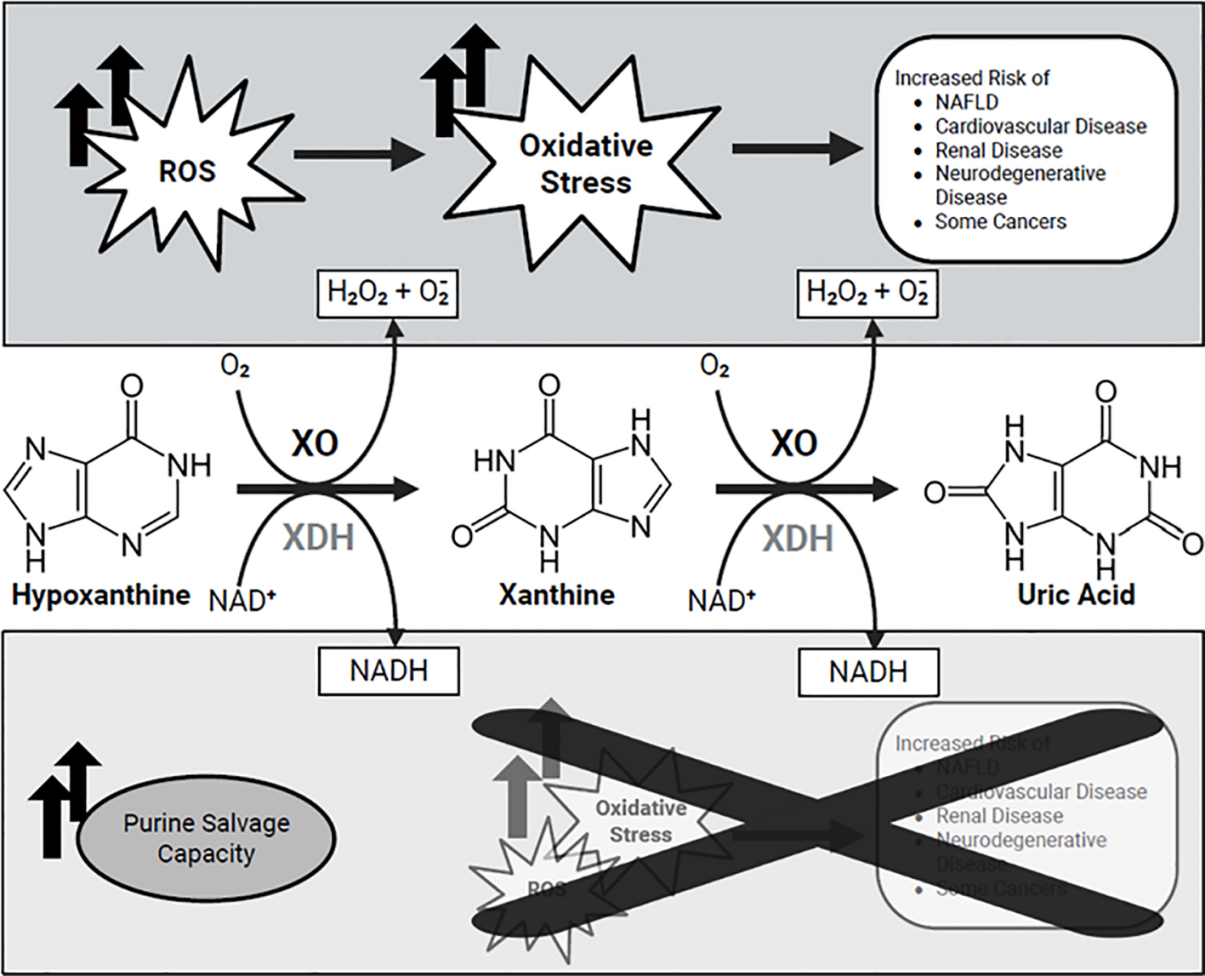
Comparison of reactions carried out by XOR proteoforms xanthine dehydrogenase (XDH) and xanthine oxidase (XO). Degradation of hypoxanthine into xanthine and further into uric acid can be carried out using XDH, employing NAD+ as the electron acceptor and generating NADH as its by product, or using XO, employing molecular oxygen as the electron acceptor and generating reactive oxygen species as a byproduct. NADH, Nicotinamide adenine dinucleotide; H_2_O_2_, Hydrogen peroxide; NAFLD, Non-alcoholic fatty liver disease; ROS, Reactive oxygen species.

XOR catalyzes the final steps of purine catabolism: the oxidation of hypoxanthine to xanthine, and xanthine to UA. XOR exists as interconvertible proteoforms, xanthine dehydrogenase (XDH) and xanthine oxidase (XO), and while both carry out the same purine conversions, they utilize different electron acceptors, thus generating different byproducts despite culminating in UA (21). XDH carries out the hypoxanthine to xanthine and the xanthine to UA reactions using NAD+ as the electron acceptor, generating NADH as its by product. Conversely, XO uses molecular oxygen as the electron acceptor, generating hydrogen peroxide and superoxides. Thus, degradation of purines resulting in identical UA concentration may result in no additional generation of reactive oxygens species (ROS) when catalyzed by XDH but may result in significant increase in ROS generation when catalyzed by XO (**Figure 2**) (21). Inconsistency in the association between UA and certain pathologies may then be the result of differences in enzymatic activity generating UA and not the UA concentration itself. This mini-review focuses on the structure, regulation, and pathological roles of XOR, with emphasis on its interaction with purinergic signaling and implications in oxidative stress-related diseases.

### Xanthine oxidoreductase

XOR is a molybdo-flavoenzyme encoded by the *XDH* gene on chromosome 2p23.1 and is initially translated as the XDH proteoform. In this state, XDH preferentially uses NAD^+^ as an electron acceptor, producing NADH as a byproduct in the degradation of purines toward UA, resulting in no additional generation of ROS (21, 22). However, once XDH is expressed, it can be converted to the XO proteoform. In this form molecular oxygen is used as the electron acceptor, resulting in generation of superoxides, and hydrogen peroxide as byproducts in the degradation toward UA. The conversion of XDH to XO can occur via a reversible oxidation reaction or an irreversible proteolytic reaction (21, 23). In conditions of mild oxidative stress, disulfide bonds may form between Cys535 and Cys992 and between Cys1316 and Cys1324 of XDH, resulting in a conformational change in the enzyme to the XO proteoform (23). Reducing agents such as dithiothreitol (DTT) can reverse this change, reverting XO to the XDH form (23).

In more extreme cases, such as hypoxia (24, 25), ischemia (24, 25), or inflammation (21), calcium-dependent proteases cleave XDH into an XO proteoform, resulting in an irreversible shift to XO activity (21). During hypoxia or ischemia, reduced oxygen results in increased thiol oxidation and the formation of disulfide bridges. Rapid oxygen reperfusion can magnify the negative effects, as the reintroduction of oxygen triggers the now-converted XO to generate additional ROS (24–26). In cases of inflammation, cytokines such as tumor necrosis factor-alpha (TNF-α) and interleukin-1beta(IL-1β) increase transcription of XDH, and inflammatory conditions promote the proteolytic, irreversible conversion of XDH to XO (27).

This interplay between XDH and XO activity determines the degree to which purine degradation contributes to redox homeostasis or leads to increased oxidative stress and injury (21). XDH activity creates a net antioxidant effect as it generates UA without generating additional ROS (22), while XO activity’s production of UA is partnered with generation of superoxide and hydrogen peroxide (22). Given that this activity is more common under conditions of inflammation and poor antioxidant defense (21, 25), a shift toward XO activity and away from XDH activity promotes excessive oxidative tissue damage (21, 25). Thus, individuals may have equivalent XOR transcription activity, XOR protein content, and UA concentrations but experience vastly different redox states. The activity of the different proteoforms, then, is of critical importance in disease contexts and likely plays some role in the inconsistent associations between UA and certain pathologies reported in previous literature (21, 22). Thus, the products of XOR activities, UA, reduced NADH from XDH and ROS from XO have wide ranging effects. On one hand there are antioxidant and neuroprotective effects of UA and generation of ATP by NADH by donation of electrons to electron transport chain (28). On the other hand, there are detrimental effects of ROS on cellular signaling, inflammation and oxidative stress (27) among many others that are described in this review.

### Purinergic signaling and XOR

Purinergic signaling is a communication system involving several nucleotides (ATP, ADP, AMP, UTP, UDP) and nucleoside adenosine (29). The purinergic signaling molecules act as ligands to two families of purinergic receptors, P1 (ADORA1, ADORA2A, ADORA2B, ADORA3) and P2 (P2X and P2Y) (30, 31). P1 receptors are specific to adenosine and P2X receptors are specific to ATP whereas P2Y are activated by ATP, ADP, AMP, and UDP. The system also involves ecto-enzymes known as ectonucleotidases, which catalyze hydrolysis reactions (32). Purinergic receptors are expressed in several cell types and are critical for signaling in processes like neuromodulation, secretion, cell proliferation, differentiation and development, etc. XOR activity and purinergic signaling are closely linked. XOR inhibition provides the purines necessary for purine salvage pathway and affects the subsequent purinergic signaling activity, and the nature of that XOR activity, be it via XO or XDH activity, impacts balance of oxidative species. (33). P1 receptors are of particular interest because the caffeine metabolite methylxanthine, one of many purine metabolites, acts as an antagonist of P1 receptors and competes with adenosine when binding to adenosine receptors (34). Similarly, inosine, a breakdown product of adenosine and a substrate of XOR, also competes with adenosine to bind to P1 receptors (35). However, caffeine and inosine actions are different, one being antagonistic and the other being an agonist to adenosine (36). Also, XOR metabolizes several steps in caffeine catabolism, and caffeine is an inhibitor of XO activity (37). The interplay between XOR and purinergic signaling is crucial for several physiological processes.

### Genetic variants in *XDH* and risk for metabolic diseases

The *XDH* gene which encodes XOR is found on human chromosome 2 and is comprised of about 60Kb, made up of 27 exons. Mutations in *XDH* result in type I and II Xanthinuria. Although individuals with these mutations may experience xanthine crystallization into renal calculi, as well as renal failure and myopathies, 50% of them remain asymptomatic. Several *XDH* genetic variants have been linked to impaired purine metabolism and related-metabolic diseases. In a study in cancer patients in Spain, rs207454 on *XDH* was linked to survival in breast cancer patients who are progesterone receptor (PGR) negative (38). The same SNP was also linked to survival in patients with gastric cancer in a study from China (39, 40). Other studies that studied *XDH* SNPs link with oxidative stress related metabolic diseases showed their important role in sepsis and acute respiratory diseases, where the authors postulated that *XDH* maybe a genetic and biochemical marker for risk outcome for these diseases (41). Others have shown that *XDH* SNPs are associated with diabetes and kidney disease (42); altered NO2^-^ substrate and O2^-^ levels (43); and blood pressure and incident hypertension (44). Overall, *XDH* polymorphisms have been implicated in higher ROS and RNS production and oxidative stress (45) and risk for related metabolic diseases. Furthermore, XDH mutations have been shown to affect its gene expression. Massimo et al. showed in COS-7 cells that the mutants Asn909Lys, Thr910Lys, Asn1109Thr, His1221Arg and Pro555Ser in *XDH* resulted in negligible levels of protein. As compared to the wild type. Likewise, XOR enzymatic activity was also significantly affected by its gene mutations (43).

### XOR in obesity, diabetes and non-alcoholic fatty liver disease

Obesity is a metabolic disorder that is characterized by excess fat accumulation leading to many other metabolic disturbances (46). The imbalance between energy intake and expenditure is key to the development of obesity. Given the close link between energy and purine metabolism, purine metabolites are often implicated in obesity (47). Serum concentrations of urate have been shown to be elevated in obesity and diabetes (48). Similarly, XOR activity is often associated with BMI, independent of UA. In a human study, BMI, smoking, alanine transaminase, triglycerides and insulin resistance as measured by HOMA-IR were independent predictors of XOR activity, which is also negatively linked to adiponectin (49). In a study of non-alcoholic fatty liver disease (NAFLD), XOR activity was increased and was positively correlated with BMI and insulin resistance (50). In a study in mice investigating the role of XOR inhibition in obesity, XOR inhibitor increased burning of lipids and activated the purine salvage pathway, thus potentially helping in weight loss (51). When individuals were divided into two categories of low and high XOR activity, the Furuhashi et al. found most of the individuals with obesity were in the higher XOR activity category (49).

### XOR in cardiovascular-renal diseases

In circulation, XOR is converted from its dehydrogenase form to oxidase form (52). In vascular dysfunction, pro-inflammatory agents such as IL-1, IL-6, and TNF-α increase transcription of XOR (53) while circulating XO binds to vascular endothelial cells, gaining stability and increasing production of ROS (54). Once this shift occurs, the superoxide radicals generated by the increased XO activity reduces bioavailability of nitric oxide (NO) which in turn impairs vasodilation and promotes oxidative damage of low density lipoprotein (LDL) cholesterol, promoting plaque formation (52, 53). In cardiac ischemia-reperfusion injuries, XDH conversion to XO is primarily via irreversible proteolysis (25) and results in a large influx of ROS when tissue is reperfused, a mechanism of great import in cases of myocardial infarction, stroke, and peripheral vascular disease (21, 24, 25). The XOR products also modulate arteriolar tone and endothelial function (55).

In cases of essential and secondary hypertension, shifts toward the XO proteoform are seen, especially in the renal vasculature (56). Hypertension risk can also be elevated with increased XOR activity which can increase inflammation, decrease endothelial NO availability, mediate endothelial dysfunction, and activate vascular remodeling (40, 57, 58). The activity of XO in these tissues promotes renal inflammation and injury (59). Inhibitors of XO, such as allopurinol and febuxostat, have been shown to reduce blood pressure and proteinuria in hypertensive and hyperuricemic models due to ROS suppression more so than the lowering of UA concentration (60, 61). The contribution of XOR to chronic kidney disease (CKD) is multifaceted. On one hand, it can increase deposition of urate crystals in the renal tubules and medullary interstitial spaces, causing gouty nephropathy, on the other hand, it can lead to glomerular and tubular injury and fibrosis through its action of increasing ROS. In patients on hemodialysis, glycemic control assumed importance as patients in glycemic control were able to manage their XOR activity and ROS production as compared to others (62). Similarly, the duration of dialysis and the type of renal replacement therapy used seem to affect XOR activity (63). In contrast, a study in rats showed that XDH is essential for kidney maturation and its absence leads to developmental issues and susceptibility to kidney damage in adulthood (64).

### XOR in neurological and neurodegenerative diseases

The brain is uniquely vulnerable to oxidative stress due to its high oxygen consumption, abundance of polyunsaturated lipids, and relatively low antioxidant capacity (65, 66). Because of this, the relative activity levels of XDH or XO are of paramount importance. In normal physiological conditions, XOR activity in the brain is low, and the predominate form is XDH, leading to no additional ROS generation (67, 68). However, neuroinflammation, ischemia, or mitochondrial dysfunction can induce shifts, both reversible and irreversible, toward XO. The resulting increase in superoxide and hydrogen peroxide can cause or exacerbate neuronal damage, cerebral aneurysms (69) and can damage the blood-brain barrier (65, 67, 68).

Oxidative stress is a well-established feature of both Alzheimer’s disease (AD) and vascular dementia (67, 70). The XDH/XO balance may modulate cerebrovascular health and neuronal integrity via redox signaling. Burrage et al. demonstrated that XO inhibition with febuxostat ameliorated cognitive deficits and improved cerebral vasodilation in a mouse model of chronic stress. These effects were not attributable to UA, suggesting that XO-derived ROS rather than UA concentration contributed to impaired neurovascular function (67). In human studies, use of inhibitors of XO were associated with lower incident dementia rates (71). These findings support the hypothesis that XO activity specifically, not just XOR activity in general or its resulting UA concentration, contributes directly to cognitive decline, which is likely through chronic ROS exposure affecting vascular and neuronal resilience.

Multiple sclerosis (MS) is a neurodegenerative disease involving inflammation of the central nervous system and demyelination of nerve cells; and ROS are heavily implicated in progression of degeneration (72). In an experimental autoimmune encephalomyelitis (EAE) model, Honorat et al. found increased XO activity within CNS lesions, along with elevated ROS. Subsequent treatment with febuxostat reduced XO activity, ROS generation, inflammatory infiltration, and clinical severity of the condition (72). This suggests that shifting from XDH activity toward XO activity contributes to oxidative stress and resultant injury in MS. Parkinson’s disease (PD) is a neurodegenerative disease characterized by dopaminergic neuron loss in the substantia nigra (73). Watanabe et al. investigated the combined administration of febuxostat and inosine in PD patients. Improvements in motor scores were observed after 8 weeks, but the mechanisms were not solely attributable to increased UA levels (74). Given febuxostat’s specificity for XO, these findings imply that XO-derived ROS may exacerbate dopaminergic neurotoxicity, and that inhibition of this XO activity may be neuroprotective. In addition, purine salvage pathway, which is augmented by XOR inhibition, is more effective in generating ATP than *de novo* synthesis (75). Thus, this pathway is crucial for brain cells and for generating adenosine, a neurotransmitter.

### XOR in cancers

Purine metabolism has been implicated in cancers as an important link between tumorigenesis and cancer progression. Battelli et al. noted that in many tumors, increased XO activity correlated with worse outcomes only when associated with high ROS load, suggesting that it is not total XOR, but ROS-generating XO activity that mediates oncogenic signaling, angiogenesis, and immune evasion. (76). Veljkovic et al. found a strong positive correlation between XO activity and prostate-specific antigen and oxidative stress (77). To underscore that it is the XO activity itself and not its resulting UA which could come about via XDH activity as well, downregulation or loss of XDH has been linked to potentiation of gastric, breast and hepatocellular cancers. Linder et al. (78) found that decreased XDH was a predictor of poor prognosis in early-stage gastric cancer (78). Similar results were provided by Chen et al. who found reduced expression of XDH and promotion of TGFB signaling in hepatocellular carcinoma cells (HCC) (79). The expression and activity of XOR in cancer varies based on the tissue of origin, with lower levels observed in gastrointestinal, colorectal, liver and breast tumors and higher levels in prostate, bladder and lung cancer as compared to non-cancerous tissues of the same type (80). Thus, XOR inhibition therapy needs to be tissue specific and should be considered carefully, particularly because XOR inhibition augments the purine salvage pathway, which may, in turn, provide more nucleotides for malignant cells (80). In some cases, increasing expression of XOR may enhance the effectiveness of chemotherapy (81), while in other cases, XOR-derived ROS have been shown to promote progression to malignancy (76). Taken together, these observations indicate that XOR has a dual role in cancer with XDH activity being associated with better oncological states while XO activity amid oxidative stress is detrimental in cancer.

### Sex and lifestyle-specific effects on XOR

The activity of XOR shows sex-specific differences. Like UA, XOR activity is higher in males than females, although the levels of XOR in blood are low (82). The association between XOR activity and related metabolic disorders such as hypertension, diabetes, fatty liver disease and kidney diseases has been shown to be significantly different between sexes across various population groups (83–86). Dietary intake of specific nutrients also affects XOR activity. High intake of diets rich in saturated fats, and fructose increase XOR activity in plasma (87). Lastra et al. (87) also showed that XO inhibition protected the mice against aortic stiffness and impaired vasorelaxation induced by Western diets. Moreover, XO overexpression promoted lipid accumulation in a *xdh* knockin mice model (88). Another lifestyle factor, physical exercise affects XOR activity. Few studies have been conducted researching this aspect of XOR and they have shown contradictory results based on the type and intensity of the physical exercise. On one hand, physically intensive marathon increased XOR activity in young men (89), on the other hand, aerobic exercise training reduced XOR activity in middle aged men and women (90). These findings show the importance of genetic as well as lifestyle factors in the variation in XOR activity.

### XOR in other disorders

Conversion of XDH to XO can result from mechanical injury and inflammation, and this conversion in keratinocytes at the site of injury results in local accumulation of ROS (91). The ROS impair healing, and chronic wounds and pressure injuries show sustained activity of XO in contrast to XDH, delaying wound closure (22). Weinstein and colleagues (91) treated diabetic wounds with XDH siRNA, resulting in reduced mRNA expression, XO activity, ROS levels, and pathological wound burden, and a 7 day acceleration in wound healing (91). XO has also been involved in exercise-induced oxidative stress. A study in rats showed an increase in XO activity in response to an exhaustive physical exercise which was ameliorated by allopurinol (XO inhibitor). The authors postulated that the increase in XO during exercise may result in increased superoxide production in the vascular bed. This was supported by another study where the authors found that XO binds to sulphated glycosaminoglycans in the vascular endothelial cells, which may promote superoxide production (92, 93). Similarly, in mice, Yisireyili et al. found that restraint stress in mice increased XOR and XO activity and ROS in visceral adipose tissue. Treatment with febuxostat (another XO inhibitor) not just reduced XO but also improved serum UA concentrations, insulin sensitivity and prothrombic state (94).

## Discussion

XO activity, beyond simply the resulting UA concentration, is implicated in pathologies in many tissues and organs in both human and animal models. Evidence suggests that this is due to the associated generation of ROS not seen when XDH catalyzes similar reactions in purine metabolism.

Inflammation, mechanical stress, hypoxia and ischemia-reperfusion all can drive the XOR enzyme from its initial XDH form toward its XO conformation, increasing the generation of superoxide and hydrogen peroxide. These resulting ROS can impair endothelial function, oxidize cellular components, and damage mitochondria. The ROS overwhelm endogenous antioxidant capacity and may impair endothelial dilatation, oxidize lipoproteins and other cellular components, and damage mitochondria. Cardiovascular (53, 56) and renal (59–61) studies demonstrate that ROS burden, rather than serum UA concentration itself, predicts dysfunction, while diabetic wounds (91), solid tumors (76), and neurodegenerative disorders (67, 72, 74) demonstrate similar effects in which high XO activity is associated with worse outcomes when controlling for UA concentration.

Despite these documented associations from many research groups covering many different pathologies, the precise mechanisms governing the favoring of XDH or XO over its counterpart remain unclear. Post-translational modifications, availability of substrate, proteolytic cleavage, and redox potential of the environment all likely contribute to this ratio. This complexity undoubtedly also plays a role in understanding the variable outcomes for different pathologies despite similar UA concentrations.

Therapeutically, inhibitors that block the molybdenum center of XO (allopurinol, febuxostat) confer benefits that exceed what would be expected from UA lowering alone. In animal and human models they restore endothelial nitric−oxide bioavailability, blunt inflammatory cell infiltration, and improve cognitive or motor performance (67, 71, 74). They have also been shown to increase central nervous system (CNS) adenosine during hypoxia potentiating its neuroprotective effects (95), in addition to increasing energy delivery in human failing hearts (96). The inhibition of XOR also increases availability of purine nucleotides and restoration of ATP levels through the salvage pathway (75).

Complicating investigation into this topic, accurately quantifying XO activity *in vivo* remains technically challenging. Epidemiological studies tend to rely on serum UA concentration or purine ratios as surrogates for enzyme activity, and so these do not capture the differences in ROS production that come from different ratios of XDH and XO activity. Future research in this area should focus not only on how targeted inhibitors like febuxostat alter outcomes, but on measurement of the relative activity of both proteoforms of XOR. The adoption of broader assays meant to specifically measure XO activity and itss oxidative products, separate from measurement of XDH activity, will be of the utmost importance in future research in this area.

Individual variability and the roles of precision medicine and precision nutrition are similarly highlighted by these factors. Genetic variability in the XOR gene, differences in capacity to balance oxidative stress, and specific context within different tissues all play into heterogeneity of outcomes observed across populations at similar UA concentrations. Thus, incorporating direct measures of XO activity into future clinical studies will allow for more accurate prediction of risk and more appropriate targeting of interventions.

In light of these findings, it is clear that elucidating the role of purine metabolism in pathological conditions must involve not only measurement of the purine metabolites themselves, but corresponding analysis of redox homeostasis. Across many pathologies, disease risk rises when the physiological context favors conversion of XDH to XO. With this in mind, seemingly contradictory epidemiological associations between UA and health outcomes are able to be understood. Future work should aim to characterize the drivers of the transition from XDH and XO and if this is carried out in a reversible manner, identify and validate biomarkers of ROS derived from XO activity, and determine whether modifying the balance of the proteoforms offers better therapeutic specificity than indiscriminate targeting of UA concentration.

## Conclusion

Research that focuses only on UA concentration cannot fully explain the divergent associations observed between purine metabolism and chronic disease. The balance between XDH and XO, the two proteoforms of the XOR enzyme, determines whether purine degradation is redox-neutral or strongly pro-oxidant. Evidence across cardiovascular, renal, dermatological, oncological and neurological fields shows that excess XO-derived ROS, rather than UA itself, is a likely mediator of tissue injury and clinical progression. Pharmacological inhibition or down-regulation of XO ameliorates pathology even when UA concentrations remain unchanged, underscoring the therapeutic relevance of the proteoform-specific mechanism. This review does not deny that UA may contribute to these pathologies; rather, it stipulates such an association exists. The central message is that inconsistencies across prior studies may arise because, while UA is relevant, it must be considered in the context of the reactions that generate it. This review does not aim to re-evaluate UA’s role per se, but to highlight that XDH and XO activity are at least as important as UA concentration. Equal UA levels can yield very different ROS byproducts depending on the XDH/XO balance, which may obscure UA’s apparent role. Future epidemiological and interventional studies should quantify XO activity directly rather than focusing on the concentration of purines themselves or estimating enzyme activity by comparing ratios of the purine metabolites.
